# Photo-crosslinking hydrogel for wound healing in a pilonidal sinus patient after open surgery

**DOI:** 10.1093/jscr/rjad152

**Published:** 2023-05-03

**Authors:** Yiheng Yang, Haojie Yang, Yilin Han, Zhenyi Wang, Changpeng Han

**Affiliations:** Yueyang Hospital of Integrated Traditional Chinese and Western Medicine, Shanghai University of Traditional Chinese Medicine, Shanghai 200437, China; Yueyang Hospital of Integrated Traditional Chinese and Western Medicine, Shanghai University of Traditional Chinese Medicine, Shanghai 200437, China; Yueyang Hospital of Integrated Traditional Chinese and Western Medicine, Shanghai University of Traditional Chinese Medicine, Shanghai 200437, China; Yueyang Hospital of Integrated Traditional Chinese and Western Medicine, Shanghai University of Traditional Chinese Medicine, Shanghai 200437, China; Yueyang Hospital of Integrated Traditional Chinese and Western Medicine, Shanghai University of Traditional Chinese Medicine, Shanghai 200437, China

**Keywords:** Photo-crosslinking hydrogel, Wound healing, Pilonidal sinus, Open surgery, Tissue integration

## Abstract

Pilonidal sinus is a chronic infectious disease with large incision and high risk of relapse after surgical management. Therefore, effective intervention strategies are urgently needed to reduce the relapse and shorten the wound healing time. Hydrogels have been widely used in regenerative medicine for its great biocompatibility, however, it remains challenging to integrate the material with wound tissues. Here, we reported a case of pilonidal sinus patient using a novel tissue integration material, Photo-crosslinking hydrogel after open surgery. A 38-year-old man with a pilonidal sinus for ˃5 years underwent open surgery. When the surgery was finished, the wound was injected with hydrogel that was irradiated with a ultraviolet light source until covered and solidified completely. Hydrogel needed to be changed 1–2 times per week. We evaluated the healing time as primary outcome and then followed up for ˃1 year to observe the relapse. The wound healed completely in 46 days after open surgery, which was shorter than that reported in other studies. Meanwhile, no recurrence was detected during follow-up. Photo-crosslinking hydrogel effectively promoted wound healing and has the potential to be easily applied in Pilonidal sinus patients after open surgery.

## INTRODUCTION

Pilonidal sinus is a chronic infectious sinus disease occurring in the soft tissue of the sacrococcygeal intergluteal fissure. It is characterized by hidden hair and tends to occur in young men. The disease is complex and easy to relapse [1]. Although varieties of treatment techniques with less tissue damage and faster healing for pilonidal sinus have been proposed, the latest guideline consider open surgery to be one of the most effective surgical techniques for pilonidal sinus, which reduces the relapse compared to the minimally invasive techniques [2]. The ineluctable result, however, is that open surgery causes larger wounds and longer healing time, which may even be accompanied by long-term inactivity [3] and increase anxiety.

Medical dressing, as a common therapy for wound healing after open surgery, mainly takes effect due to preventing bacterial entry, reducing pain and accelerating wound epithelialization [4]. Meanwhile, an ideal dressing can fit the shape and size of the wound well and do not require frequent replacement to avoid wasting medical resources. Hydrogel, a popular type of medical dressing, is often used in biomedical applications because of the similar mechanical behavior of hydrogels with that of living tissues and their good compatibility and ability of hydrogels to swell in water [5]. Despite various hydrogels have been tested for wound healing, it remains challenging to integrate synthetic material with surrounding tissues because there are limited surgical methods and adhesives that can be used for bonding or fixing the material [6–9].

Herein, We developed a novel Photo-crosslinked hydrogel system, Photo-induced imine-crosslinked (PIC) hydrogel to realize material-tissue integration. PIC was photoisomerized by o-nitrobenzyl alcohol, resulting in aldehyde groups synchronizing with amino groups on the tissue surface and reaction to form imine-crosslinked hydrogels with strong tissue adhesion. An imine-based photogelation process can achieve intimate biomaterial-tissue integration [10]. PIC hydrogel not only has the advantages of convenient operation，biocompatibility, strong tissue adhesiveness and precise temporal and spatial controllability of the gelation process, but also overcomes the shortcomings of similar hydrogels [10]. The crosslinking can closely integrate the hydrogel material with the wound surface through chemical bonds, promoting the wounds healing. Material-tissue integration can provide stable biofixation, reduce the risk of infection, and facilitate the wound repair process [6, 11].

Animal experiments have confirmed that the hydrogel can achieve fast sealing and adhesive hemostasis on moist wounds. The hydrogel quickly stopped bleeding, and the blood loss after sealing was significantly less than that in the control group [6]. Rat model of dorsal skin defect, the hydrogel group showed faster healing of wound within 7 days than the other groups, the wound healing time was shortened by a third [11]. Rapid hemostasis of porcine heart and carotid hypertension bleeding points was achieved by photosensitive hydrogel, and the wound was steadily sealed in only 20 s without suture [11]. Experiments in both rat and pig models of oral mucosal disease well confirmed the conclusion that hydrogels can promote surgical incision repair and accelerated healing [12]. The preliminary test results show that the hydrogel is not only safe and easy to use, with no adverse reactions but also that its tissue adhesion and integration characteristics can effectively bond and seal the wound. The test results again confirm that the use of the hydrogel can avoid secondary injuries caused by traditional sutures, such as pain, bleeding and scarring, thus saving medical resources. This new technique has been shown to be effective and safe in previous relevant animal experiments, but clinical data are lacking.

In our study, we included a 38-year-old male patient with pilonidal sinus who was treated with hydrogel during surgery. Depending on the wound healing time, it was confirmed that Photo-crosslinking hydrogel shortened the healing time and achieved better medical efficacy.

## SURGICAL TECHNIQUE

### Photo-crosslinking hydrogel application

**Figure 1:**
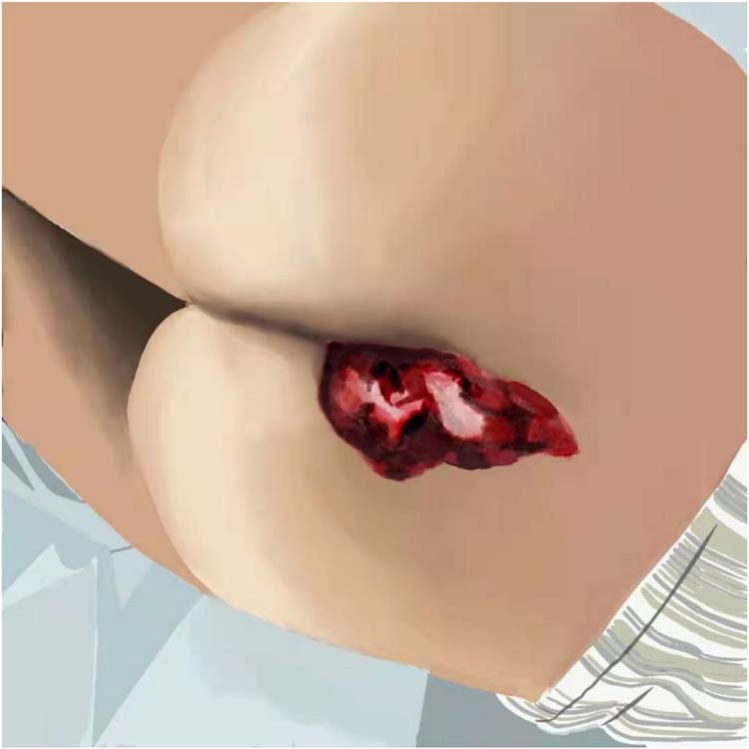
Depicts hydrogel application process.

**Figure 2:**
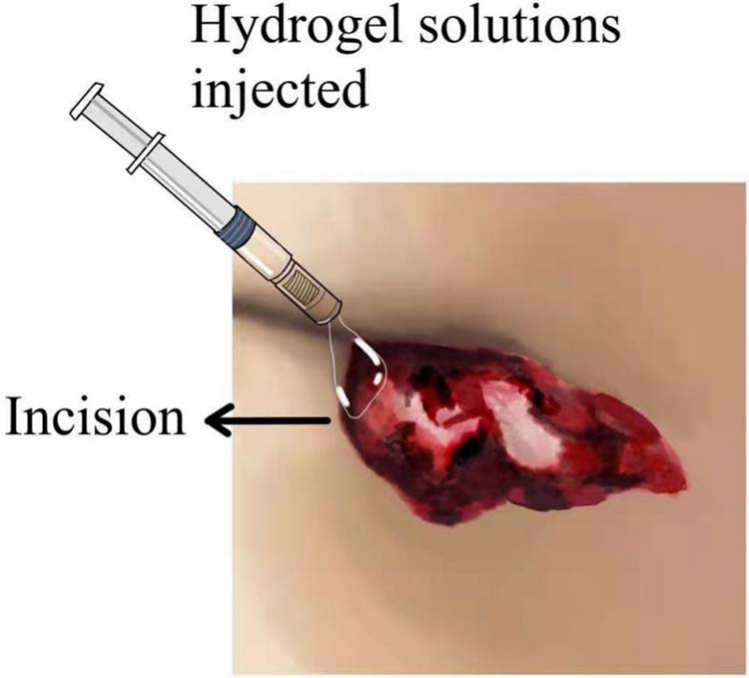
Depicts hydrogel application process.

**Figure 3:**
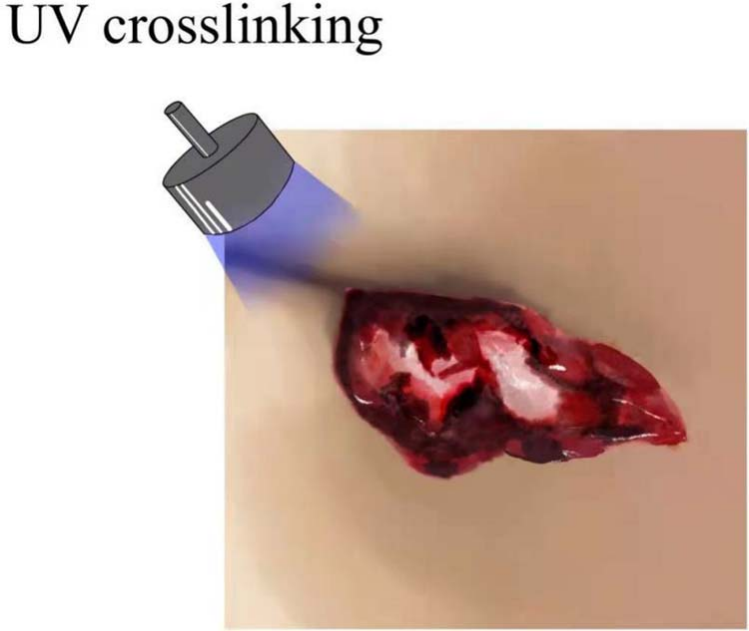
Depicts hydrogel application process.

When the surgery was finished, the prepared photo-crosslinking hydrogel was injected into the wound until completely covered. Then the ultraviolet light source was irradiated for 20–30 s until the hydrogel was solidified and combined with wound tissues (Figs 1–3 depicts hydrogel application process).

### Participant

The patient underwent all preoperative preparations and signed an informed consent form to be treated with hydrogel; the surgeon also talked to the patient’s family, who signed the informed consent form for the operation and other related procedures. In addition, the patient and his family were informed about the precautions after the operation. The other details are the same as conventional open sinus surgery.

**Figure 4:**
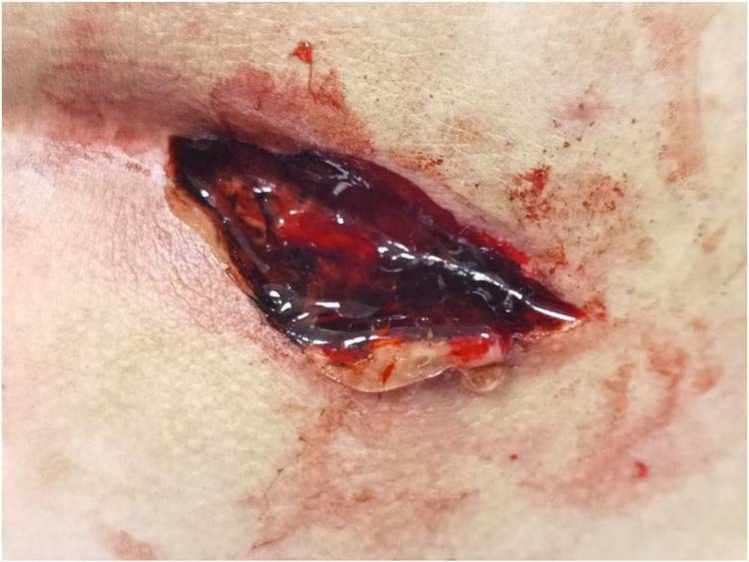
Depict the wound was sealed with hydrogel.

During the operation, it was found that a hard mass of 15 cm was palpable from the sacrococcygeal to the anal margin. The pathologic tissue was removed with a diamond-shaped incision downward to the fascia underneath. The wound was kept completely open and covered with hydrogel (Fig. 4 described the wound covered with hydrogel).

## RESULTS

**Figure 5:**
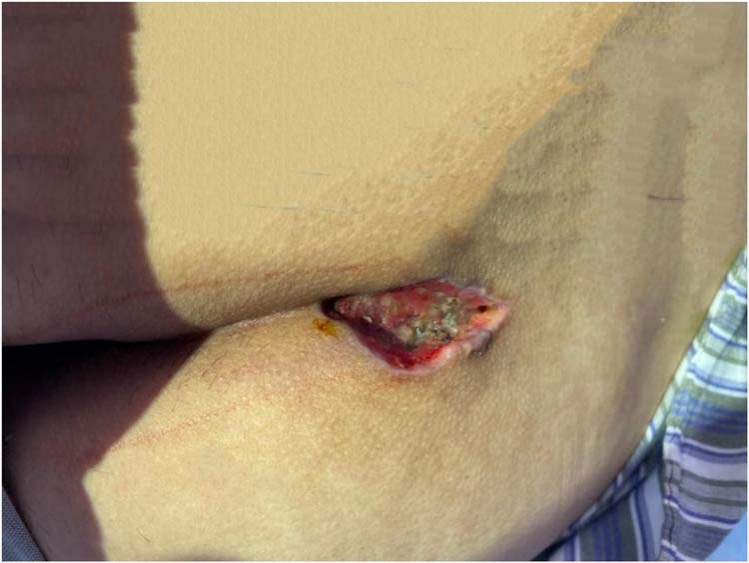
Depict wounds on discharge.

**Figure 6:**
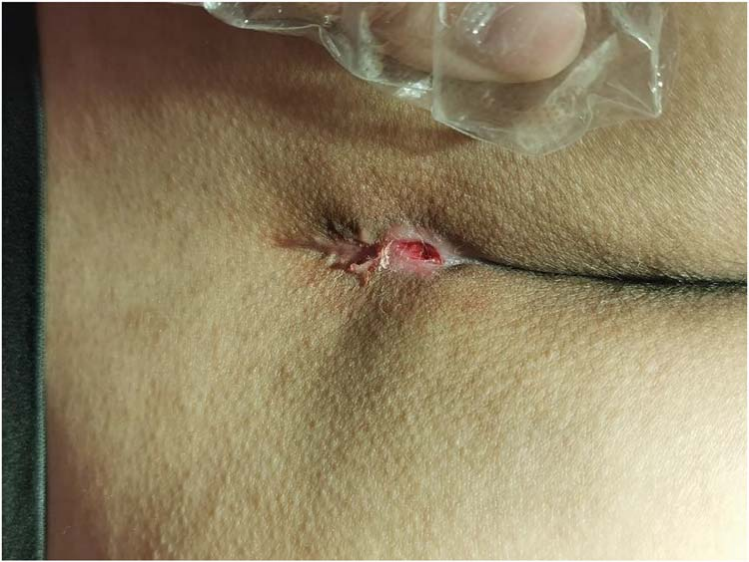
Depict wound healing.

To further evaluate the progress of wound healing, we tracked the wound healing progress over different periods. The patient was hospitalized for a total of 14 days. The wound basically recovered when the patient was discharged from the hospital (Fig. 5 showed that the wound reduced when the patient was discharged from the hospital). The patient was instructed to follow-up twice a week with outpatient visits in the first 2 weeks after discharge from the hospital, and follow-up once a week for 1 month. The wounds closed and healed completely in one and a half months (Fig. 6 showed the completely recovered wound after 46 days the patient underwent the surgery). In this case, the wound healing time was 46 days, which was shorter than the reported healing time of open surgery 54.64 ± 14.87 days and 62.20 ± 5.18 days [13, 14]. Meanwhile, no recurrence was detected during follow-up.

## DISCUSSION

Although the optimal surgical procedure is controversial [15], the goal of surgical treatment is to eradicate suppuration, to achieve healing and limit the risk of recurrence as much as possible to alleviate the suffering of patients [16]. As the latest guidelines recommend, open surgery is one of the most effective techniques with the lowest risk of relapse for pilonidal sinus. Therefore, the larger wound and longer healing time are ineluctable challenges for anorectal doctors. Complaints such as pseudo-wound healing, relapse, exudation, pain, delayed bleeding usually occurs.

Here, we applied a hydrogel adhesive technology, this technique enables combines fast, non-radical, photo-crosslinking and covalent anchoring on the tissue surface with steps, activating the hydrogel within seconds. The ideal hydrogels will have many properties, including being highly safe, aseptic, highly compatible and so on. Traditional hydrogels mainly include methyl α-cyanoacrylate and fibrin glue. The traditional hydrogels has poor adhesion and integration abilities, as it cannot adhere or integrate with wet tissue surfaces [17], and has poor biocompatibility and mechanical properties. PIC hydrogels have the advantages of convenient operation, biocompatibility, strong tissue adhesiveness, precise spatial and temporal controllability of gelation process, and overcome the shortcomings of similar hydrogels.

In our study, the wound healing time with hydrogel was 46 days, which was at least 1 week shorter than other similar procedures, for example, related reports 54.64 ± 14.87 days, 62.20 ± 5.18 days and 64.75 ± 6.50 days [13, 14, 18]. Meanwhile, PIC only needs to be changed 1–2 times per week, which is convenient for use and avoid wasting medical resources.

In conclusion, we reveal that PIC has good efficacy and convenience in promoting wound healing. We speculate that photoinitiated crosslinked hydrogels may be a promising material for wound repair. Next, we will conduct a large sample clinical research to optimize management, extend follow-up time and comprehensively evaluate the advantages of using hydrogels. This may provide a novel solution for the treatment of such diseases in the future.

## CONSENT TO PARTICIPATE

Written informed consent was obtained from individual or guardian participants.

## Data Availability

All data supporting the findings of this study are available within the article or from the corresponding authors upon reasonable request.

## CHINESE CLINICAL TRIAL REGISTRY

Registration number: ChiCTR2100053776.
